# Dental Management of a Pediatric Patient With Allergic Bronchial Asthma: A Case Report Highlighting Allergen Avoidance and Anxiety Control

**DOI:** 10.7759/cureus.102367

**Published:** 2026-01-27

**Authors:** Nikita Saini, Prateek Mishra, Elanangai E, Elampavai Elangovan, Aishwarya Jethi

**Affiliations:** 1 Dentistry, Govt. Civil Hospital Ranjhi, Jabalpur, IND; 2 Dentistry, Govt. Seth Govind Das (Victoria) District Hospital, Jabalpur, IND; 3 Prosthodontics, NSVK Sri Venkateshwara Dental College and Hospital, Bengaluru, IND; 4 Oral and Maxillofacial Pathology, NSVK Sri Venkateshwara Dental College and Hospital, Bengaluru, IND

**Keywords:** allergen avoidance, anxiety control, bronchial asthma, dental management, latex hypersensitivity, pediatric dentistry

## Abstract

Bronchial asthma is among the most prevalent chronic respiratory disorders in children and presents distinct challenges during dental care due to the risk of acute bronchospasm triggered by psychological stress, exposure to allergens, and certain dental materials or medications. Meticulous modification of routine dental protocols is therefore essential to ensure patient safety. This case report describes the dental management of a 13-year-old female patient with allergic bronchial asthma who presented with acute pain and swelling in the posterior tooth region. A multidisciplinary approach was adopted, including prior consultation with the treating pulmonologist, comprehensive medical evaluation, and implementation of preventive strategies to minimize known triggers. Particular emphasis was placed on anxiety reduction, early morning appointments, continuous monitoring of vital parameters, and implementation of a latex-free protocol. As part of the latex-free protocol, nitrile gloves were used for routine handling, and vinyl gloves were modified and employed as an alternative to the conventional rubber dam to achieve effective isolation while eliminating latex exposure. Dental treatment was successfully completed under local anesthesia without adrenaline, with emergency medications readily available. The patient remained clinically stable throughout the procedure, with no respiratory complications. This case highlights the importance of individualized treatment planning and demonstrates that simple, cost-effective modifications, such as the use of modified vinyl gloves for isolation, can facilitate safe and effective dental care in pediatric patients with allergic bronchial asthma, particularly in resource-limited settings.

## Introduction

Bronchial asthma is a chronic inflammatory disorder of the airways characterized by reversible airflow limitation and bronchial hyperresponsiveness, resulting in episodic wheezing, breathlessness, and coughing. It is one of the most common chronic diseases affecting children worldwide and remains a major contributor to disability-adjusted life years in the pediatric population, underscoring its substantial global health burden [1-3]. In India, childhood asthma prevalence demonstrates considerable variability, ranging from approximately 2% to 18.2%, which may be attributed to differences in environmental exposures, urbanization, diagnostic practices, and reporting methodologies across regions [4].

The dental management of pediatric patients with asthma necessitates special precautions, as asthma can be influenced by psychological factors such as stress, anxiety, and sadness, as well as by exposure to environmental irritants or allergens, exercise, infection, and certain pharmacologic agents commonly used in dental practice, all of which may precipitate acute asthmatic exacerbations [1,5,6]. Additionally, hypersensitivity to materials such as latex poses further challenges, increasing the risk of allergic reactions during dental procedures and necessitating the use of alternative materials [7]. Consequently, careful treatment planning, anxiety control, awareness of material sensitivity, and readiness to manage medical emergencies are essential components of safe dental care in this population [1]. This case report describes the comprehensive dental management of a pediatric patient with allergic bronchial asthma, emphasizing individualized treatment planning, behavioral management, strict allergen avoidance, and emergency preparedness to ensure safe and effective dental treatment.

## Case presentation

**Figure 1:**
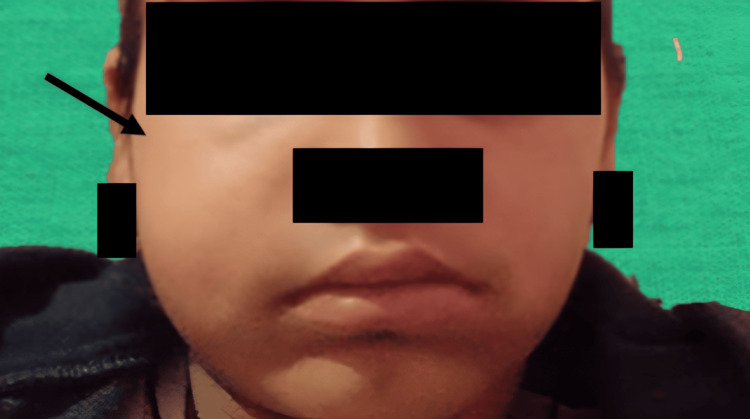
Preoperative extraoral photograph showing facial swelling in the right posterior maxillary region

A 13-year-old female patient presented to the Department of Pediatric and Preventive Dentistry with a chief complaint of pain and swelling in the upper right posterior tooth region for the past two days (Figure 1).

The patient had a documented history of allergic bronchial asthma and had been under medical management for the condition for the past four years. She reported approximately seven asthmatic episodes over the last two years, primarily triggered by exposure to dust and psychological stress. At the time of the dental consultation, the patient was asymptomatic from a respiratory standpoint, with no signs of wheezing or respiratory distress.

**Figure 2:**
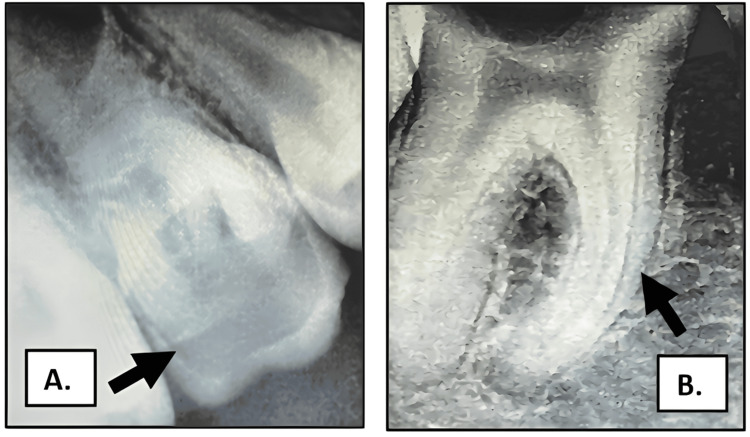
Preoperative radiographs showing deep occlusal caries in teeth 16 and 46 (A) Deep occlusal caries in tooth 16 (maxillary right first molar). (B) Deep occlusal caries in tooth 46 (mandibular right first molar).

On intraoral examination, multiple carious teeth were observed. Deep occlusal caries involving teeth 16 and 46 were noted, with tenderness on percussion (Figure 2).

Prior to dental intervention, a detailed medical evaluation and written consent were obtained from the patient’s treating pulmonologist. Medical records indicated that the patient had been asthmatic since early childhood, with symptoms exacerbated by exposure to dust and smoke. She had been using a salbutamol inhaler for four years and was currently prescribed fluticasone furoate (100 mcg) with vilanterol (25 mcg) inhalation powder, along with montelukast-levocetirizine (Montek-LC). Baseline vital parameters were within normal limits, including oxygen saturation of 97%, pulse rate of 70 beats per minute, and blood pressure of 124/71 mmHg. A comprehensive allergy panel revealed hypersensitivity, reinforcing the need for careful elimination of potential triggers during dental treatment.

**Figure 3:**
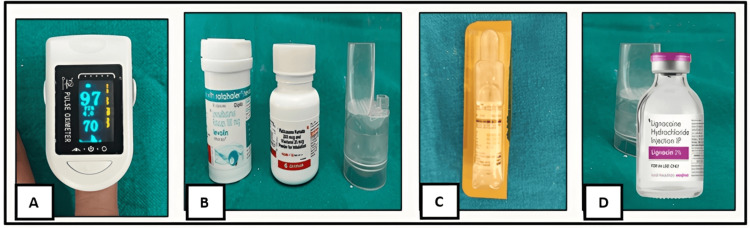
Monitoring equipment and medications prepared prior to dental treatment (A) Pulse oximeter. (B) Levosalbutamol Rotacaps 100 mcg, fluticasone furoate 100 mcg, and vilanterol 25 mcg inhalation powder. (C) Intravenous theophylline. (D) Local anesthesia without adrenaline.

Dental treatment was planned with particular emphasis on anxiety control and maintenance of a trigger-safe clinical environment. Early morning appointments were scheduled to reduce fatigue and minimize the risk of asthma exacerbation. A calm and reassuring approach was adopted throughout the appointment to reduce psychological stress. All procedures were scheduled during a symptom-free period. Emergency medications required for management of an acute asthmatic episode, including inhaled bronchodilators and intravenous theophylline, were kept readily available. Continuous monitoring of oxygen saturation was performed using a pulse oximeter throughout the procedure. Local anesthesia without adrenaline was administered to avoid potential respiratory stimulation (Figure 3).

**Figure 4:**
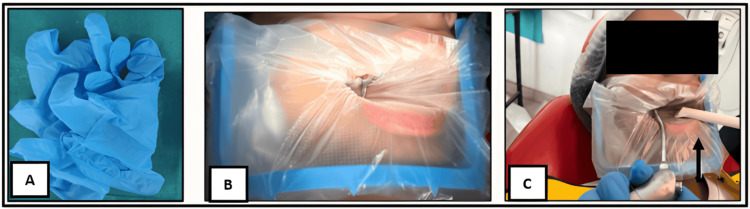
Latex-free materials and modified isolation technique used during dental treatment (A) Nitrile, disposable, powder-free, latex-free gloves. (B) Vinyl gloves modified as an alternative to a rubber dam. (C) Latex-free plastic dental oral evacuation tips.

In view of the patient’s hypersensitivity and the risk of latex-induced reactions, a latex-free clinical protocol was implemented. Nitrile, powder-free, latex-free gloves were used by the dental team. As an alternative to the conventional rubber dam, vinyl gloves were modified and used as a dental dam for isolation, eliminating latex exposure while maintaining adequate moisture control. Additionally, latex-free plastic oral evacuation tips were employed (Figure 4).

**Figure 5:**
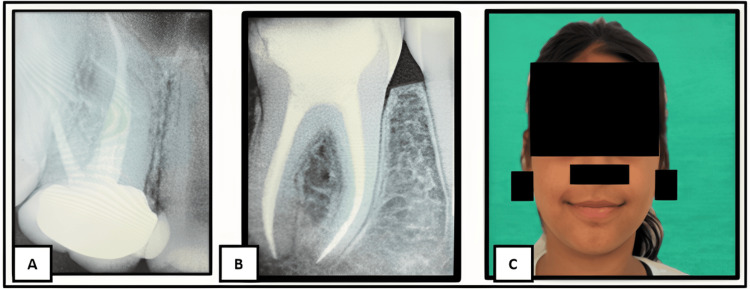
Postoperative radiographs of teeth 16 and 46 with resolution of extraoral swelling (A) Postoperative radiograph of tooth 16. (B) Postoperative radiograph of tooth 46. (C) Postoperative extraoral photograph.

The use of gutta-percha was carefully considered due to reports of chemical similarity with latex, which may trigger allergic reactions in susceptible individuals. Endodontic treatment of the affected teeth was completed successfully (Figure 5).

Preoperative and postoperative intraoral, extraoral, and radiographic evaluations were performed to assess treatment outcomes. The patient remained clinically stable throughout the procedure, with no episodes of bronchospasm or respiratory compromise, and the postoperative course was uneventful.

## Discussion

When dental treatment is provided during an asymptomatic phase and with appropriate precautions, safe and effective care can be achieved in the outpatient setting. In the present case, prior consultation with and written consent from the treating pulmonologist played a crucial role in treatment planning. Baseline assessment of vital parameters and confirmation of disease control ensured that dental procedures were performed during a stable phase of the condition. Similar recommendations have been emphasized in the literature, highlighting the importance of evaluating asthma severity, current medication use, and recent exacerbation history before initiating dental treatment [1,5].

Psychological stress and dental anxiety are well-recognized triggers for asthma exacerbations in children [7]. In this patient, anxiety reduction was prioritized through early morning appointments, a calm clinical environment, and continuous reassurance throughout the procedure. These measures likely contributed to the absence of intraoperative respiratory distress. Behavioral management and effective dentist-patient communication are particularly important in pediatric patients, as emotional factors can significantly influence airway reactivity [1,5,7].

Minimization of allergen exposure was another key aspect of management. Latex hypersensitivity is a well-recognized concern in susceptible individuals, and repeated exposure may increase sensitization and the risk of allergic reactions [6,8]. Accordingly, a latex-free clinical protocol was implemented throughout the procedure. Nitrile, powder-free gloves were used for routine handling to prevent inadvertent latex exposure, while modified vinyl gloves were employed as an alternative to the conventional rubber dam to achieve adequate isolation and moisture control. Although rubber dam isolation is widely regarded as the gold standard in restorative and endodontic procedures because of its superior moisture control, visibility, and patient safety, its routine use may pose a risk in individuals with suspected or confirmed latex hypersensitivity [5,9,10].

In the present case, the modified vinyl glove provided effective isolation without exposing the patient to latex, serving as a practical and economical alternative in a pediatric patient with heightened airway sensitivity and dental anxiety. While this technique may not offer the same rigidity or seal as a standard rubber dam, it allowed safe completion of the procedure without compromising clinical outcomes. Nitrile gloves were not adapted for isolation purposes, as their use in this manner may not be cost-effective, particularly in resource-limited clinical settings. This report describes the management of a single pediatric patient and therefore has inherent limitations regarding generalizability. The modified vinyl glove isolation technique, while effective in this case, may not provide isolation equivalent to conventional rubber dam systems in all clinical situations. Further studies with larger sample sizes are needed to evaluate the efficacy, reproducibility, and long-term outcomes of such alternative isolation techniques in pediatric patients with asthma or latex hypersensitivity. Additionally, the use of latex-free plastic oral evacuation tips further reduced the potential for allergen exposure. These tailored modifications underscore the importance of flexible adaptation of standard dental protocols in pediatric practice, where airway reactivity, anxiety, and material sensitivity demand heightened caution to ensure patient safety [1].

Emergency preparedness remains an essential component of dental care for patients with asthma [1,5]. In this case, emergency medications, including inhaled bronchodilators and intravenous theophylline, were kept readily available, and continuous monitoring using a pulse oximeter was employed throughout the procedure. Local anesthesia without adrenaline was selected to avoid potential respiratory stimulation. Local anesthetics containing vasoconstrictors have traditionally been avoided in pediatric asthmatic patients due to concerns related to sulfite preservatives such as sodium metabisulfite. However, evidence suggests that these agents can be used safely in selected cases, although caution is advised because of potential additive effects with β₂-agonists, which may result in cardiovascular effects such as palpitations, elevated blood pressure, and arrhythmias [1].

Material-related hypersensitivity is another important consideration in patients with latex allergy. Although gutta-percha is historically derived from tree latex, modern commercially purified gutta-percha is generally considered biologically safe, and true IgE-mediated cross-reactivity with latex is rare and remains controversial. Nevertheless, given the patient’s documented hypersensitivity and the potential for material-related reactions reported in isolated cases, a cautious approach was adopted during endodontic treatment. Care was taken to prevent extrusion of gutta-percha beyond the apex, and the patient was closely monitored throughout the procedure for any signs of adverse allergic response [10].

## Conclusions

Dental treatment in pediatric patients with allergic bronchial asthma can be performed safely when thorough medical assessment, multidisciplinary coordination, and individualized treatment planning are employed. Key components of successful management include anxiety control, careful scheduling, continuous monitoring, and strict avoidance of known allergens. This case highlights the clinical value of modifying standard dental protocols, particularly through the use of latex-free materials. The adaptation of vinyl gloves as an alternative to the conventional rubber dam provided effective isolation, eliminated the risk of latex exposure, and offered a practical, cost-effective solution in settings where latex-free rubber dam systems may be unavailable or unaffordable. Such flexible, patient-centered approaches are especially relevant in pediatric dental practice and contribute to safe, comfortable, and efficient care for children with asthma.
